# Preoperative High, as well as Low, Platelet Counts Correlate With Adverse Outcomes After Elective Total Hip Arthroplasty

**DOI:** 10.5435/JAAOSGlobal-D-20-00049

**Published:** 2020-09-01

**Authors:** Rohil Malpani, Patawut Bovonratwet, Michael G. Clark, Taylor D. Ottesen, Michael R. Mercier, Jonathan N. Grauer

**Affiliations:** From the Department of Orthopaedics and Rehabilitation, Yale School of Medicine, New Haven, CT (Mr. Malpani, Dr. Bovonratwet, Mr. Ottesen, Mr. Mercier, and Dr. Grauer), and Vanderbilt University School of Medicine, Nashville, TN (Clark).

## Abstract

Preoperative laboratory studies are often obtained as part of the workup for surgeries such as total hip arthroplasty (THA). An increasing need exists to be able to identify patients at risk for adverse outcomes. Thus, metrics that correlate with postoperative adverse events and readmissions are increasingly important to optimize patient care. The implications of varying abnormal platelet counts, especially on the high end of the spectrum, have yet to be assessed in large, multicenter patient populations. This study aims to risk stratify THA patients with varying preoperative platelet counts to address these questions. The purposes of this study were to (1) evaluate cutoffs for normal versus abnormal platelet counts for patients undergoing THA by using postoperative complications data and (2) assess the correlation of such values with readmission data using the National Surgical Quality Improvement Program database.

**Methods:**

Patients who underwent elective primary THA were identified in the 2011 to 2015 National Surgical Quality Improvement Program database. Risk of 30-day perioperative complications was calculated as a function of preoperative platelet counts. Based on the risk criteria, patients were categorized into the following three groups: normal platelet counts, abnormally low platelet counts, and abnormally high platelet counts. Multivariate analyses were performed to compare 30-day postoperative complications, readmissions, surgical time, and length of hospital stay between these populations.

**Results:**

The current study identified 86,845 THA patients. Using the relative risk threshold of 1.5, platelets counts were divided into abnormally low (≤142,000/mL) and abnormally high (≥417,000/mL) categories. Higher rates of any, major, and minor adverse events and hospital readmission were associated with both the abnormally low and high platelet cohorts.

**Conclusion:**

This study suggests that preoperative high, as well as low, platelet counts are correlated with perioperative complications after THA, including hospital readmissions. Patients with these laboratory findings warrant further attention with possible preoperative and postoperative optimization.

Over the past decades, the demand for total hip arthroplasty (THA) has grown significantly because of the aging population and the successes of this intervention.^1^ Consequently, THA has become one of the most common orthopaedic procedures in the United States.^2-4^ Concurrently, the value of each part of care continues to be scrutinized. For THA, this is exemplified by the development of Bundled Payments for Care Initiative that reimburses healthcare providers based on episode of illness rather than a pay-per-service strategy.^5^ In an effort to increase physician and hospital accountability, pre-established metrics have been outlined for key outcomes such as readmissions and complications.^6^

Routine preoperative laboratory study data may be useful in the management and risk stratification of THA patients. Previous investigations evaluating preoperative laboratory values as a predictive tool for general surgery have investigated postoperative outcomes because of low platelet counts for those with platelet disease processes such as immune thrombocytopenia^7^ or preoperative laboratory values and outcomes among aggregated cohorts of “noncardiac surgery” populations, not differentiating for orthopaedic procedures.^8^ One such study found thrombocytopenia and thrombocytosis to be positively correlated with postoperative complications in noncardiac procedures.^9^

For studies focused specifically on orthopaedic procedures, such as THA, thrombocytopenia has been shown to be inversely correlated with postoperative blood loss in a small cohort of hip surgery patients (n = 438).^10^ Another single-institution cohort study with limited patient numbers (n = 544) found preoperative laboratory values, including platelet counts, to hold little predictive power.^11^

Owing to the increased number of THAs being performed and the need to identify patients at risk for adverse outcomes, measures that correlate with postoperative complications and readmissions are becoming increasingly important to identify for the healthcare team. In light of the fact that previous orthopaedic studies evaluating preoperative platelet counts for the THA population are limited, inconsistent, and underpowered, the current study uses the American College of Surgeons National Surgical Quality Improvement Program (NSQIP) database to address these issues.

The purposes of this study were to (1) evaluate cutoffs for normal versus abnormal platelet counts for patients undergoing THA by using postoperative complications data and (2) assess the correlation of such values with readmission data using a large, national patient sample.

## Methods

### Database and Patient Population Details

#### Overview of the National Surgical Quality Improvement Program Database and Inclusion Criterion

The NSQIP database contains over 250 variables for each surgical case from over 500 participating healthcare institutions across the United States.^12^ These variables capture patient demographics, preoperative comorbidities, perioperative adverse outcomes, and readmission data for 30 days after the procedure. Our institutional review board granted an exemption for studies using this data set.

Patients who underwent primary THA between 2011 and 2015 were identified using the Current Procedural Terminology code 27130. This range of data years was considered because the NSQIP database in years 2011 onward contain data on occurrence of readmission within 30 days of the procedure.

**Figure 1:**
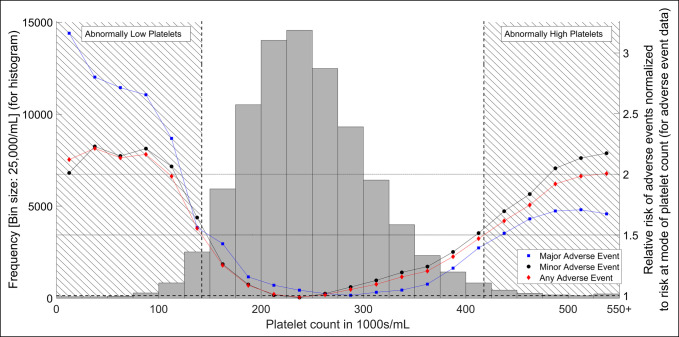
Histogram demonstrating the platelet count and plot of adverse event relative risk as a function of platelet count for total hip arthroplasty patients. Left y-axis refers to the histogram in the figure. Horizontal lines denote relative risks of 1.5 and 2 as reference lines. Right y-axis refers to the line and scatter plot of adverse event data. Data are presented as a moving average and are normalized to the risk at the mode of the histogram giving us relative risks for different platelet counts. Vertical dashed lines and cross-hatching denote the different platelet categories. Squares represent major adverse events, circles represent minor adverse events, and diamond represents any adverse events.

Preoperative platelet counts were obtained, and a histogram of platelet counts was charted. This was overlaid with adverse event frequency data as a function of platelet count (Figure 1). Using the any adverse risk threshold of 1.5, patients were divided into three categories based on preoperative platelet counts (further explained in the results section): normal, abnormally low, and abnormally high platelet counts.

#### Exclusion Criterion and Details on Missing Data

The exclusion criterion included the presence of preoperative fractures, neoplasms, infections, and/or emergency/trauma cases. In addition, any cases with revision codes were excluded (Current Procedural Terminology codes 27134, 27137, and 27138).

After the initial patient selection, a small proportion (<1.00%) of patients had missing data for studied patient demographic characteristics. These patients were dropped from the study. After this, a further 4.63% of the THA patients identified had missing preoperative platelet count values and were dropped. A previous study in the orthopaedic patient population has shown that dropping such low numbers of patients (leading to a complete case analysis) should not skew results.^13^

#### Patient Preoperative Characteristics

Patient characteristics and demographics such as age, sex, height, weight, functional status before surgery, American Society of Anesthesiologists (ASA) classification, preoperative platelet count, diabetes mellitus status, and smoking status were directly abstracted from the NSQIP database. Body mass index (BMI) was calculated from the height and weight data (mass in kilograms divided by the squared height in meters). ASA score was used as a marker of comorbidity, in accordance with the previous literature.^14,15^

#### Perioperative Outcomes

The NSQIP database tracks patient outcomes for 30 days postprocedure. Individual perioperative complications were aggregated to identify groups of adverse events: any, major, and minor adverse events.

The occurrence of a major adverse event was defined as the occurrence of any of the following: death, return to operating room, sepsis/septic shock, unplanned intubation, ventilator use >48 hours, stroke/cerebrovascular accident, cardiac arrest, acute renal failure, pulmonary embolism, deep vein thrombosis, wound infection, and readmission within 30 days. The occurrence of a minor adverse event was defined as the occurrence of any of the following: wound dehiscence, urinary tract infection, pneumonia, progressive renal insufficiency, and transfusion. The occurrence of any adverse event was defined as the occurrence of a major or minor adverse event.

Operating time and length of stay (LOS) were also extracted from the NSQIP database. Operating time was measured in minutes, whereas LOS was measured in days (days between procedure and discharge).

### Statistical Analysis

#### Platelet Count/Adverse Event Plot Construction

Adverse event data were overlaid on a histogram of platelet counts. The adverse event data were normalized by adverse event risk at the mode of the platelet count histogram, yielding relative risk ratios. The adverse event data were converted to a moving average (five bin step size) to reduce granularity and noise in the data.

Although the platelet counts were found to fall into a normal distribution, the relative risk of adverse events fell into a U-shaped curve with higher risk for adverse events at the extremes of the platelet count spectrum. At either end of the plot, the points at which the relative risk increased beyond a relative risk of 1.5 were used to divide the patients into normal, abnormally low, and abnormally high platelet count groups. Similar curves were generated for any, major, and minor adverse events and three specific adverse outcomes (transfusions, thromboembolic events [defined as the occurrence of a pulmonary embolism or of a deep vein thrombosis], and hospital readmissions).

#### Univariate Analysis

Initially, patient characteristic and demographic factors of those with the three different platelet categories (low, normal, and high) were assessed and compared with univariate analyses. Similarly, perioperative adverse outcomes (any, major, and minor adverse events and readmissions, operating times, and LOS) were analyzed similarly.

Chi-squared tests or Fisher exact tests were used for categorical variables, whereas two-tailed two sample Student t-tests using groups were used for continuous variables. Statistical significance was set at α = 0.05 for both.

#### Multivariate Analysis

Logistic regressions were performed for the different adverse outcomes types in both the abnormally low and abnormally high platelet cohorts relative to the normal platelet cohort. The regressions were controlled for age, sex, BMI, ASA score, and functional status. Statistical significance was set at α = 0.05, and 95% confidence intervals are reported.

Statistical analysis was performed in Stata version 13.1 (StataCorp, LP).

## Results

### Patient Population

**Figure 2:**
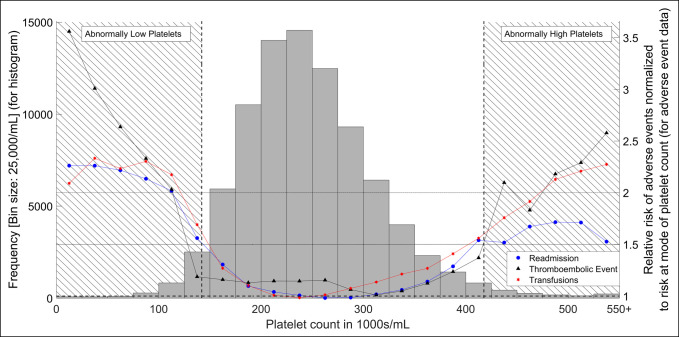
Histogram demonstrating the platelet count and plot of hospital readmissions, transfusions, and thromboembolic events as functions of platelet count for total hip arthroplasty patients. It is similar to Figure 1, but the legend is different. Circles refer to readmissions, triangles refer to thromboembolic events, and asterixes refer to transfusions.

Eighty-six thousand eight hundred forty-five THA patients were identified based on the inclusion and exclusion criteria. The platelet count distribution and adverse outcome data are shown in Figures 1 and 2. As described in the methods, the division of low and high platelet counts was made based on “any adverse event” crossing over the 1.5 relative risk threshold and were defined as low platelets if platelet count was ≤142,000/mL and high platelet count if platelet count was ≥417,000/mL.

Using the above-defined thresholds for low and high platelet counts, 82,617 patients (95.13%) were determined to have a normal platelet count, 2,748 patients (3.16%) were determined to have an abnormally low platelet count, and 1,480 patients (1.70%) were determined to have an abnormally high platelet count.

### Comparison of Preoperative Characteristics Between Different Platelet Groups

Compared with the normal platelet count cohort, the abnormally low platelet count cohort had a higher percentage of older patients (*P* < 0.001), men (69.51% versus 44.52%, *P* < 0.001), dependent functional status (*P* < 0.001), ASA scores ≥ 3 (*P* < 0.001), and diabetic patients (*P* < 0.001) (Table 1).

**Table 1:** Demographics of Patients Undergoing Total Hip Arthroplasty Organized by Platelet Category

Type	Normal Value	Abnormal Value Low	Univariate	Abnormal Value High	Univariate
Platelet Count	142-417 (1,000 s/mL)	≤142 (1,000 s/mL)	*P* Value	≥417 (1,000 s/mL)	*P* Value
Total Cases (N = 86,845) (%)	82,617 (95.13%)	2,748 (3.16%)		1,480 (1.70%)	
Age			**<0.001**		**<0.001**
18-54	14,904 (18.04%)	382 (13.90%)		335 (22.64%)	
55-64	24,877 (30.11%)	734 (26.71%)		*436 (29.46%)*	
65-74	*25,560 (30.94%)*	*846 (30.79%)*		400 (27.03%)	
≥75	17,276 (20.91%)	786 (28.60%)		309 (20.88%)	
Sex			**<0.001**		**<0.001**
Male	36,782 (44.52%)	*1,910 (69.51%)*		339 (22.91%)	
Female	*45,835 (55.48%)*	838 (30.49%)		*1,141 (77.09%)*	
BMI			0.106		**<0.001**
<25	16,890 (20.44%)	515 (18.74%)		*479 (32.36%)*	
25-30	*28,032 (33.93%)*	*965 (35.12%)*		455 (30.74%)	
30-35	21,000 (25.42%)	725 (26.38%)		298 (20.14%)	
>35	16,695 (20.21%)	543 (19.76%)		248 (16.76%)	
Functional status (before surgery)			**<0.001**		**<0.001**
Independent	*80,960 (97.99%)*	*2,645 (96.25%)*		*1,424 (96.22%)*	
Partially dependent	1,598 (1.93%)	100 (3.64%)		55 (3.72%)	
Totally dependent	59 (0.07%)	3 (0.11%)		1 (0.07%)	
ASA			**<0.001**		**<0.001**
1	3,474 (4.20%)	65 (2.37%)		33 (2.23%)	
2	*45,960 (55.63%)*	1,020 (37.12%)		*741 (50.07%)*	
3	31,833 (38.53%)	*1,530 (55.68%)*		678 (45.81%)	
≥4	1,350 (1.63%)	133 (4.84%)		28 (1.89%)	
Diabetes			**<0.001**		**0.027**
Insulin	2,081 (2.52%)	145 (5.28%)		49 (3.31%)	
Noninsulin	*7,172 (8.68%)*	*326 (11.86%)*		*148 (10.00%)*	
Smoker	10,862 (13.15%)	381 (13.86%)	0.274	310 (20.95%)	**<0.001**

ASA = American Society of Anesthesiologists classification, BMI = body mass index

Italic indicates the most common subgroup.

Bolding indicates statistical significance at *P* < 0.05.

Compared with the normal platelet count cohort, the abnormally high platelet count cohort had a higher percentage of younger patients (*P* < 0.001), women (77.09% versus 55.48%, *P* < 0.001), lower BMI values (*P* < 0.001), dependent functional status (*P* < 0.001), ASA scores ≥ 3 (*P* < 0.001), and smokers (*P* < 0.001) (Table 1).

### Univariate Adverse Outcomes Analysis

In the plot of adverse outcomes versus platelet counts, the increase in relative risk at both ends of the platelet spectrum were noted (Figures 1 and 2). This suggests correlation of abnormal platelet counts with adverse outcomes. Furthermore, this increase in the relative risk was exacerbated with more extreme values of lower/higher platelet counts.

Compared with the normal platelet group, both the abnormally high and low patient groups were associated with a higher likelihood of any, major, and minor adverse events (all *P* < 0.001) (Table 2). Both the abnormal platelet cohorts were also associated with a higher risk of readmissions as well (*P* < 0.001). In addition, longer hospital LOS was associated with both abnormally low and abnormally high platelet values (*P* ≤ 0.001). Operating time was not associated with adverse events for either the abnormally low or the abnormally high platelet cohort (Table 2). Please keep in mind that one patient might have multiple separate adverse events within one adverse event category. Because of this, the sum of the subcategories for adverse events (eg, death, stroke etc.) is higher than the total events listed under the particular adverse event category (eg, major adverse events).

**Table 2:** Platelet Category Versus Perioperative Adverse Events for Total Hip Arthroplasty

Type	Normal Value	Abnormal Value Low	Univariate	Abnormal Value High	Univariate
Platelet Count	142-417 (1,000 s/mL)	≤142 (1,000 s/mL)	*P* Value	≥417 (1,000 s/mL)	*P* Value
Total No. of Cases (N = 86,845)	82,617 (95.13%)	2,748 (3.16%)		1,480 (1.70%)	
Adverse event	**12,836 (15.54%)**	**694 (25.25%)**	**<0.001**	**383 (25.88%)**	**<0.001**
Major adverse event	**4,161 (5.04%)**	**235 (8.55%)**	**<0.001**	**119 (8.04%)**	**<0.001**
Death	110 (0.13%)	15 (0.55%)		4 (0.27%)	
Sepsis/septic shock	245 (0.30%)	17 (0.62%)		7 (0.47%)	
Unplanned intubation	132 (0.16%)	13 (0.47%)		4 (0.27%)	
Ventilator > 48 hr	52 (0.06%)	11 (0.40%)		2 (0.14%)	
Stroke	68 (0.08%)	2 (0.07%)		3 (0.20%)	
Cardiac arrest	54 (0.07%)	5 (0.18%)		1 (0.07%)	
MI	186 (0.23%)	15 (0.55%)		2 (0.14%)	
Acute renal failure	38 (0.05%)	2 (0.07%)		1 (0.07%)	
Thromboembolic event (PE, DVT)	494 (0.60%)	17 (0.62%)	0.935	16 (1.08%)	**0.018**
Wound infection	948 (1.15%)	63 (2.29%)	**<0.001**	22 (1.49%)	0.226
Return to OR	1,596 (1.93%)	85 (3.09%)		51 (3.45%)	
Readmission	2,819 (3.41%)	155 (5.64%)	**<0.001**	86 (5.81%)	**<0.001**
Minor adverse event	**9,799 (11.86%)**	**561 (20.41%)**	**<0.001**	**309 (20.88%)**	**<0.001**
Wound dehiscence	85 (0.10%)	7 (0.25%)		7 (0.47%)	
UTI	816 (0.99%)	33 (1.20%)		15 (1.01%)	
Pneumonia	252 (0.31%)	23 (0.84%)		8 (0.54%)	
Progressive renal insufficiency	72 (0.09%)	14 (0.51%)		2 (0.14%)	
Transfusion	8,869 (10.74%)	517 (18.81%)	**<0.001**	288 (19.46%)	**<0.001**

Type	Normal Value	Abnormal Value Low	Univariate	Abnormal Value High	Univariate
Platelet Count	142-417 (1,000 s/mL)	≤142 (1,000 s/mL)	T-test *P* Value	≥417 (1,000 s/mL)	T-test *P* Value
Total No. of Cases (N = 86,845)	82,617 (95.13%)	2,748 (3.16%)		1,480 (1.70%)	
Operating time (min)			0.138		0.068
Mean	93.29	94.17		94.88	
SD	40.75	42.14		41.29	
LOS (d)			**<0.001**		**<0.001**
Mean	2.85	3.31		3.09	
SD	3.97	3.84		2.28	

LOS = length of stay, MI = Myocardial Infarction, PE = Pulmonary Embolism, DVT = Deep Vein Thrombosis, OR = Operating Room, UTI = Urinary Tract Infection

Bolding indicates statistical significance at *P* < 0.05.

### Multivariate Adverse Outcome Analysis

**Figure 3:**
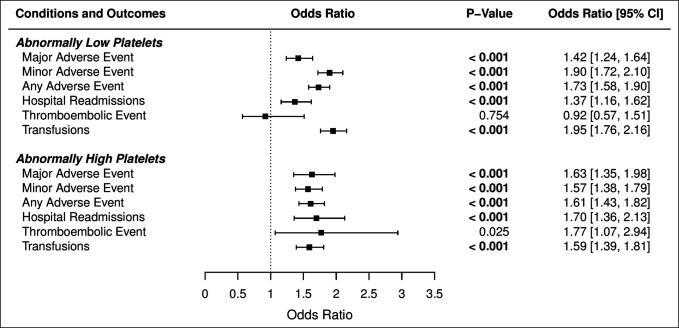
Forest plot and multivariate analysis demonstrating the abnormally low and high platelet categories for various postoperative adverse events. Contains details on multivariate analysis (logistic regression) on adverse event and readmission data for the different platelet categories and the forest plot.

Based on the platelet cutoff values defined above, multivariate analysis for THA revealed the abnormally high platelet cohort to be associated with a higher likelihood of adverse events (any [OR = 1.64, *P* ≤ 0.001], major [OR = 1.67, *P* ≤ 0.001], and minor [OR = 1.61, *P* ≤ 0.001] adverse events) and a higher risk of hospital readmission (OR = 1.74, *P* ≤ 0.001) as an independent risk factor (Figure 3).

The abnormally low platelet cohort was also associated with a higher likelihood of any (OR = 1.75, *P* ≤ 0.001), major (OR = 1.44, *P* ≤ 0.001), and minor (OR = 1.92, *P* ≤ 0.001) adverse events and a higher risk of readmission (OR = 1.38, *P* ≤ 0.001) as an independent risk factor (Figure 3).

## Discussion

THA is a common and effective surgery for patients affected by hip degeneration that is not sufficiently relieved by nonsurgical interventions.^16^ Regression models predict that the demand for primary THA will increase by 174% between 2005 and 2030.^17^ As the demand for THA increases, measures predictive of surgical outcomes have become increasingly valuable to physicians and healthcare institutions. The present study sought to determine whether preoperative platelet counts were correlated with postoperative outcomes in patients who have undergone primary THA.

The methods used in this analysis defined a normal platelet count to be between 142,000 and 417,000/mL, which closely approximates the 150,000 to 400,000/mL range commonly described in the literature.

Patients in the abnormally low platelet group were older and more likely to be male than patients in the normal platelet group. The opposite was true in the abnormally high platelet group: patients were younger and more likely to be female. These findings are consistent with previous population-based studies showing higher platelet counts in women than in men^18,19,20,21^ and decreased platelet counts with increasing age.^19,20^ Compared with the normal platelet group, patients in both abnormal platelet groups had less independent functional status, a measure that has been shown to be predictive of 30-day postoperative morbidity in orthopaedic surgery.^22^ Both abnormal platelet groups had higher rates of diabetes than the normal platelet group, consistent with the literature describing abnormal platelet values in diabetes.^23,24^ The abnormally high platelet group had a larger fraction of smokers than the normal group, consistent with the literature reporting increased platelet counts in smokers.^25^ Patients in the abnormally high platelet count group tended to have a lower BMI than those in the normal group, in contrast to the literature reporting positive relationships between BMI and platelet count.^26,27^ This may suggest that the lower BMI in the abnormally high platelet count group may be a result of other factors besides the elevated count itself. In addition, both abnormal platelet groups had increased ASA scores relative to the normal group.

Patients in the abnormal platelet count groups were significantly more likely to experience major adverse events, minor adverse events, any adverse event, hospital readmission, and longer postoperative hospital stays. In the low abnormal value group, the major adverse events with the greatest increase in frequency compared with patients with normal platelet counts were return to the operating room, superficial infection, and death. In the high abnormal value group, these events were return to the operating room, thromboembolic events, and organ space surgical site infections. Platelets play important roles in inflammatory processes,^28^ suggesting that abnormal platelet counts could mediate susceptibility and response to infection. For both abnormal groups, the minor adverse event with greatest increase in frequency relative to the normal group was postoperative transfusion, as could be expected. Abnormally low platelets were associated with a higher risk of wound infections (*P* < 0.001), whereas abnormally high platelets were not (*P* = 0.226).

The pronounced “U” shape of the moving average data in Figure 1 indicates that as platelet count becomes more abnormal, the frequency of major, minor, and any adverse events increase. These results, alongside the multivariate odds ratio analysis in Figure 3, suggest that abnormal platelet counts as defined in this study are strongly associated with postoperative adverse events and readmissions.

This strong association between low platelet counts and postoperative adverse events and readmission rates presents a significant impetus to clinicians for identifying solutions to increase platelet counts with the aim of converting an at-risk patient to the normal platelet range and lower their risk profile. The literature offers multiple examples of nutritional therapy that can aid in increasing platelet counts. In a human cell in vivo model, a bioactive fraction extracted from *Psidium guajava* (common guava) was shown to induce thrombopoietin production and increase platelet counts.^29^ Furthermore, in a murine model, an extract made from *Carica papaya* (common papaya) leaves was associated with a significant increase in platelet counts, compared with controls.^30^ In addition, both plants have been used to prevent thrombocytopenia in humans suffering from dengue fever as well.^31^

These results have important implications for hospital cost efficiency. The present study demonstrates that for patients undergoing THA, abnormal platelet counts are associated with longer hospital stays and higher readmission rates, both of which pose an economic burden to patients and healthcare systems. Figure 2 in particular provides a close look at the risk of readmission as a function of the particular patient's platelet count allowing for risk stratification.

The strengths of the present study include the large sample size and the quality of the data obtained from the NSQIP database.^32^ The NSQIP database is particularly well suited for surgical outcomes research. The results of the current study will be useful to patients, healthcare providers, and health systems for preoperative risk stratification and cost reduction purposes.

Limitations of the study include the lack of follow-up data greater than 30-days postoperation and unavailability of THA-specific outcome measures such as drain outputs and patient satisfaction information. In addition, there are lack of surgical data such as surgical approach used that can be confounders. Furthermore, the reason for the elevated or lowered platelets cannot be determined from the data set but that there is often not a definitive etiology. This study also does not evaluate patient-reported quality of life metrics because of the lack of data.

## Conclusion

Preoperative platelet count is an independent risk factor for postoperative adverse events, hospital readmission, and length of hospital stay in patient undergoing THA. Our data indicate that the frequency of adverse events generally increases as platelet count becomes more abnormal in both the low and the high directions. These findings should be useful to providers to identify at-risk populations and optimizing care.
